# Treatment of a fractured humerus and partially torn supraspinatus tendon in a professional boxer with liquid platelet-rich fibrin and heat-coagulated albumin gel: a case report

**DOI:** 10.1186/s13256-024-04458-7

**Published:** 2024-03-23

**Authors:** Torbjörn Ogéus

**Affiliations:** Stockholms led- & smärtklinik, 11424 Stockholm, Sweden

**Keywords:** Humerus fracture, Fracture healing, Delayed union, Supraspinatus, Tendon, PRF, ALB-PRF, PRP

## Abstract

**Introduction:**

Fractures with delayed healing can be a serious complication, especially for athletes depending on quick return to sports. To our knowledge, no cases of increased healing of delayed union fractures with autologous biomedical interventions have been reported.

**Case presentation:**

A 33-year-old Swedish professional boxer with a fractured humerus with a delayed union and a partially torn supraspinatus tendon was treated with injections of liquid platelet-rich fibrin and heat-coagulated albumin gel. He recovered almost completely from both injuries in only 1 month and could return to professional boxing in 3 months.

**Conclusion:**

This case raises the hypothesis that liquid platelet-rich fibrin and heat-coagulated albumin gel may be an effective, minimally invasive intervention in fractures with a delayed union. Further research is required to test this theory.

## Introduction

Delayed union of fractures are bone fractures that fail to heal in the expected time frame. A delayed fracture bears the risk of turning into a nonunion fracture when healing has little expectation of spontaneous healing [1], and can be a serious complication for the affected patient, especially for athletes depending on quick return to sports. Fractures with delayed healing in the proximal shaft of the humerus occurs in about 5% following closed treatment and should be considered for surgery when failing to demonstrate evidence of progressive healing on consecutive radiographs taken 6–8 weeks apart [2]. Partial thickness rotator cuff tears are common, with a high prevalence among athletes. Different surgical procedures are usually suggested [3]. To our knowledge, no cases of increased healing of a fracture with a delayed union have been reported with autologous biomedical interventions.

A number of modalities have been researched to aid in the healing of proximal humerus fractures (PHF), such as high-energy extracorporeal shockwave therapy [4], surgical fixation [5], and physiotherapeutic rehabilitation; despite the amount of studies in the area, no high-quality or moderate-level evidence supports surgical intervention over rehabilitation when considering the long-term outcome after 1 year and 2 years of follow-up [6].

## Case presentation

We present the case of a 33-year-old Swedish professional boxer, with a 7-week-old traumatic proximal humerus fracture and a partially torn supraspinatus tendon, which upon presentation at the clinic, had no radiographic signs of fracture healing after 4 weeks. Radiographic examination again after 7 weeks of closed treatment still did not show evidence of healing. He was under some stress since he had an important boxing match scheduled and missed important training to prepare for the upcoming match.

## Method

The injured supraspinatus tendon was confirmed with ultrasound and both the fracture and the supraspinatus tendon were treated with autologous platelet injections at the clinic.

Various types of autologous platelet injections have been proposed to treat tendons and ligaments. Some studies have indicated a positive effect on inflammation and tendinopathy in general; however, the effect of injected platelets on the injured tendon could require multiple injections and there is no standard protocol for treating tendons [7]. One way to extend the effect of injected platelets is to heat a liquid platelet-poor plasma (PPP) layer before mixing it with platelets concentrated in a separate centrifugation, thus extending the resorption properties of heated albumin (albumin gel) that leads to a release of growth factors over an extended period of time (ALB-PRF) [8].

Recent studies have shown that, in addition to treating tendons and ligaments, ALB-PRF has a wide variety of possible applications within regenerative medicine. ALB-PRF has been shown to have interesting properties in osteogenesis and has been used successfully in dentistry in grafting processes and bone healing [9]. In animal studies, evidence has been published that both solid PRF and liquid PRF holds a potent antiinflammatory capacity and reduces osteoclastogenesis [10], as well as decreases inflammation in mesenchymal cells [11].

The use of platelet injections and its effect in enhancing fracture healing has been studied, however, the amount of studies are few and the results vary, and more research is needed according to a review published in 2021 [12].

The fractured humerus of this patient and the injured supraspinatus tendon were injected two times during 1 week. The first injection was a concentrated platelet-rich fibrin (C-PRF) [13] injection of 2 ml (2 ml in each injection site, 4 ml in total), centrifuged at 2000×*g* for 8 minutes on a horizontal centrifuge. The following injection 1 week later consisted of 2.5 ml (in each injection site) of ALB-PRF, 2000×*g* for 8 minutes on a horizontal centrifuge, and the albumin layer was heated according to the ALB-PRF protocol (75° for 10 minutes). In the last step, the heat-coagulated albumin was mixed with the remaining C-PRF to create ALB-PRF. All injections were performed with ultrasound guidance.

Eccentric training of the treated supraspinatus tendon was commenced 1 week after the last injection.

## Results

**Fig. 1:**
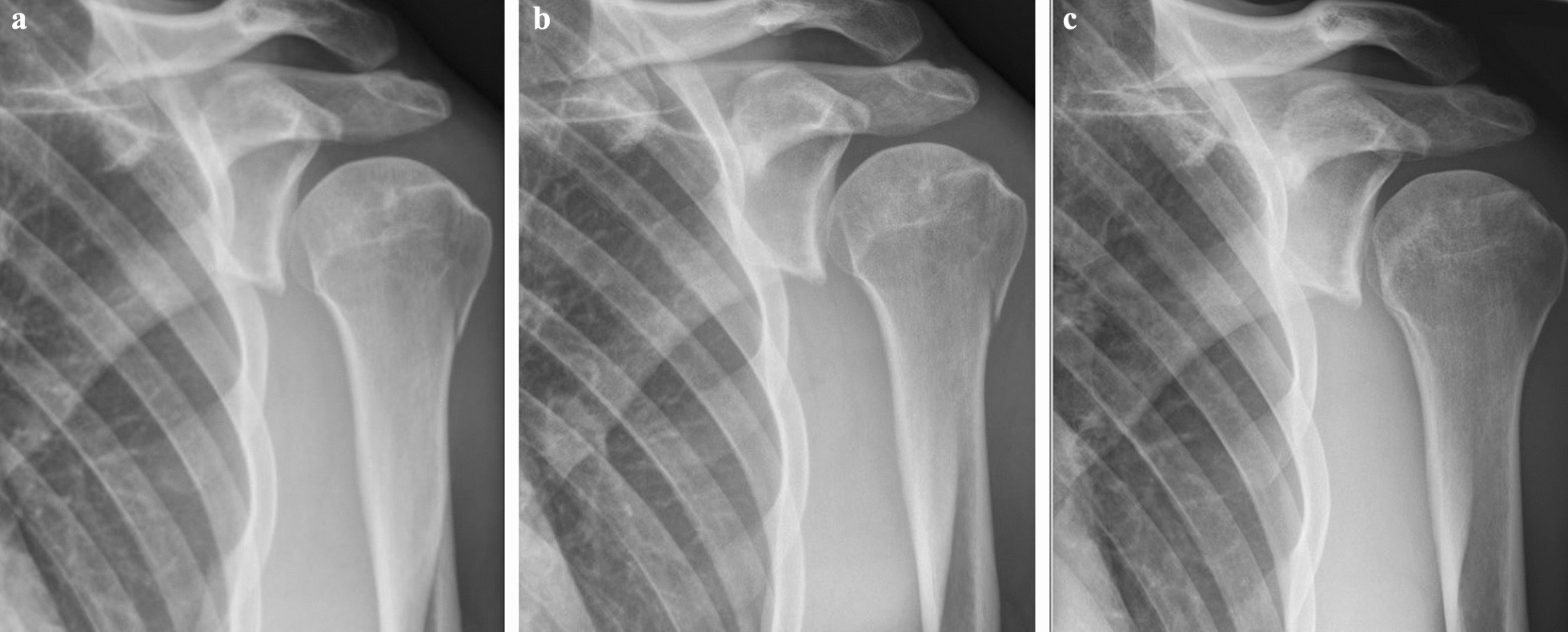
Radiographic pictures of the delayed union fracture and the healing process 18 days after the start of the treatment (**a**); 34 days between first and second picture (**b**); 18 days between second and third picture (**c**)

**Fig. 2:**
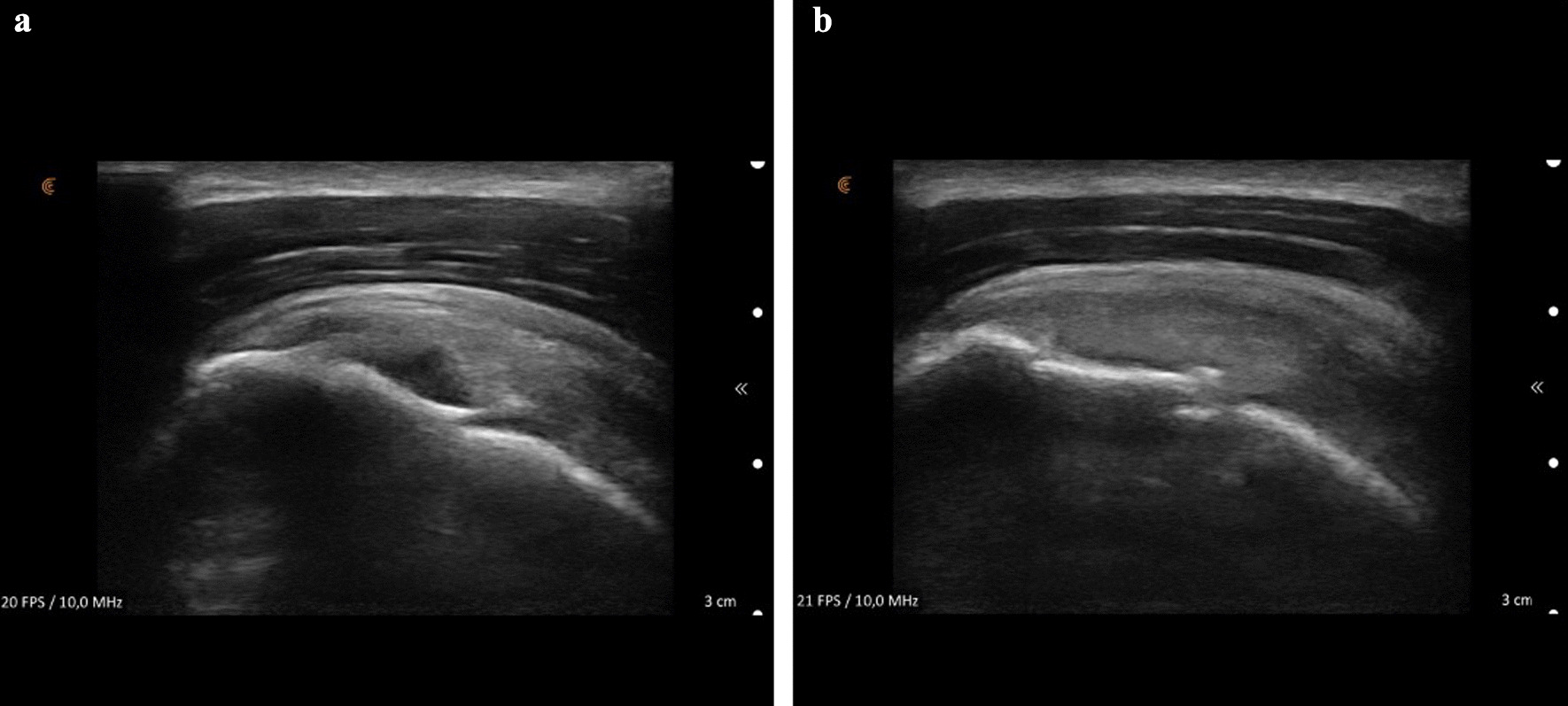
Ultrasound picture of the supraspinatus tendon of the left shoulder. First picture was taken before the treatment, showing a partial tear (**a**). The second picture was taken 18 days after the first treatment (**b**)

A total of 18 days after the start of treatment, radiographic examination showed almost complete healing of the treated proximal humerus fracture (Fig. 1). The treated supraspinatus tendon also showed clinical signs of healing, and healing was confirmed by ultrasound examination (Fig. 2). He returned to training in 1 month and returned to professional boxing in 3 months. No relapses have been reported and he is still active in his boxing career a year after treatment.

## Discussion

In this case, we presented a professional boxer with a fracture of the proximal humerus with a delayed union, combined with a painful a partial tear of the supraspinatus tendon.

Most fractures usually heal over time with conservative treatment, and arguably the natural course of healing might have been enough for the patient in this report in the long run. However, fractures with a delayed union confirmed after 6–8 weeks have little chance of spontaneous healing. The patient in the case started treatment 7 weeks after the fracture with extraordinary results, both clinically and visually on radiographic examination and ultrasound. The patient in this case was physically very active and depended on his body to earn his living. The novel PRF and ALB-PRF treatments came after the failure of conventional conservative therapies. ALB-PRF has been used in dentistry and guided bone regeneration (GBR) with some success, and studies in other fields of medicine for future applications have been warranted [14]. According to a recent review, ALB-PRF is proposed as a substitute for collagen barrier membranes in various clinical applications, such as guided tissue/bone regeneration [15].

As an alternative to surgical intervention and continued conservative treatment in fractures with a delayed union, autologous injection therapies are minimally invasive and associated with very few risks and complications, and might therefore be considered in specific cases; especially in combination with tendon or ligament injuries that might be associated with traumatic fractures in sports medicine.

The case presented herein describes a successful novel use of PRF and ALB-PRF for a proximal humerus fracture with delayed union and a supraspinatus tear, thus providing us insight into an alternative intervention for patients in whom the standard conservative treatment has failed or as an option to surgical intervention.

While the available data from studies thus far remains weak, especially regarding human studies, the potential within the field of regenerative medicine, however, is huge. Further research on PRF and ALB-PRF for the treatment of fractures with a delayed union in a larger patient group are indicated to optimize the treatment protocol further and test the theory in full. The capacity both PRF and ALB-PRF have to decrease mesenchymal cell inflammation and osteoclastogenesis might be important keys in the healing process of bone fractures that needs further studies.

## Conclusion

This case raises the hypothesis that liquid platelet-rich fibrin and heat-coagulated albumin gel may be an effective, minimally invasive intervention in fractures with a delayed union. Further research is required to test this theory.

## Data Availability

All data generated or analyzed during this study are included in this published article [and its supplementary information files]. Radiographic and ultrasound images are stored at the clinic and is available for review by the Editor-in-Chief of this journal.
